# Furanic Polymerization Causes the Change, Conservation and Recovery of Thermally-Treated Wood Hydrophobicity before and after Moist Conditions Exposure

**DOI:** 10.3390/polym15010221

**Published:** 2022-12-31

**Authors:** Bengang Zhang, Mathieu Petrissans, Anelie Petrissans, Antonio Pizzi, Baptiste Colin

**Affiliations:** 1 LERMAB, I.U.T. Hubert Curien, University of Lorraine, 7 Rue Fusillés Résistance, 88000 Epinal, France; 2 LERMAB-ENSTIB, University of Lorraine, 27 Rue Philippe Seguin, 88000 Epinal, France

**Keywords:** wood, heat treatment, hydrophobicity, wettability, Whilhelmy method, color change, chemical mechanisms, furans generation, CP MAS ^13^C NMR, MALDI

## Abstract

The Whilhelmy method of contact angle, wood thermal properties (TG/DTG), infrared spectroscopy, etc. was used to define the hydrophobicity of heat-treated beech and fir wood at increasing temperatures between 120 °C and 300 °C. By exposure to wet conditions during 1 week, the hydrophobic character obtained by the heat treatment remains constant heat-treated. Heat induced wood hydrophobation, was shown by CP MAS ^13^C NMR and MALDI ToF mass spectrometry to be mainly caused by furanic moieties produced from heat-induced hemicelluloses degradation. This is caused by the acid environment generated by the hydrolysis of the hemicelluloses acetyl groups. Furfural polymerizes to linear and branched oligomers and finally to water repellent, insoluble furanic resins. The water repellent, black colored, cross-linked polymerized furanic network is present throughout the heat-treated wood. Wood darkening as well as its water repellency due to increasing proportions of black colored furanic resins increase as a function of the increase with treating temperature, becoming particularly evident in the 200 to 300 °C treating temperature range.

## 1. Introduction

The thermal degradation of wood makes it possible to transform wood properties. New properties are obtained allowing wood to be more suited for outdoor use. Heat-treated wood is more dimensionally stable; it resists fungal attack, and it does become hydrophobic and less hygroscopic than untreated wood. These transformations are well known and allow for the industrialization and marketing of such a product [1,2,3,4,5,6,7,8,9,10,11,12,13,14,15,16,17,18,19,20,21,22,23,24,25,26,27,28,29].

On the subject of the change in wettability, very few studies exist in the literature [1,2,3,4,5,6]. Moreover, these rare studies only note the change in wetting, before and after heat treatment, without investigating different treatment temperatures and without offering any supporting explanation by chemical analysis for such an altered behavior. Finally, these studies use the sessile drop method, which is unsuitable for a porous and anisotropic support, such as wood, as the drop is absorbed during the measurement.

Recently, work by Endo et al. [7] updated the instability of the heat-treated wood’s new conferred properties. The hygroscopicity of a wood heat-treated at 120 °C does indeed decrease, in agreement with the literature [7]. However, after exposure to a humid atmosphere for 1 week, this property is affected. The lower hygroscopicity obtained by the heat treatment is reduced by the wet treatment [7]. Bekhta and Niemz found that blackening is generally accelerated when the heat treatment temperature exceeds approximately 200 °C [8]. Sivonen et al. also found that when the heat treatment temperature exceeds 200 °C, the change in wood’s chemical properties is more significant [9]. Heat treatment of beech wood at different temperatures results in different changes in the chemical composition [10,11,12]. This observation is of interest for the marketing of the product, which can then easily lose its new properties. 

In the research work presented here, the interest was to study the evolution of the wettability (contact angle at the advance) according to the intensity of treatment (temperature, time) and this by using the more correct and reliable Wilhelmy method. This is based on a tensiometric force balance, which makes it possible to know the angle of contact at the advance and at the retreat. These angles characterize the wetting hysteresis. The results are more accurate and validated on wood [3,4,13,14,15,16]. 

Previous studies of chemical variation based on FTIR are found in the literature [17,18,19,20,21,22,23,24,25,26,27,28,29,30] as well as with other techniques [31,32,33,34]. Though FTIR is a valid technique, it is not suitable to determine what really happens and what is formed chemically during the hydrophobization reaction of wood. Thus, the interest focused on the study of the mass losses the TGA/DTG, IR, 13C NMR spectra and MALDI ToF spectra to try to correlate the variation in wettability with the chemical modifications of wood at the molecular level. The interest was to try to link the variation of the contact angle either to the removing of -OH groups due to the degradation of hemicelluloses or to other modifications of the wood constituents network. A further aim of this study was to determine if the gain in hydrophobicity due to the wood thermal treatment was affected or not by a wet/moist treatment as in other authors’ previous work [7] and if the original hydrophobicity would or would not be recovered after the wet/moist treatment.

## 2. Materials and Methods

TGA/DTG (Thermogravimetric Analysis, TGA; Differential Thermogravimetry, DTG) (NETZSCH, STA 449 F3 Instruments, Weimar, Germany) and FTIR (Fourier Transform Infrared Spectroscopy) (Perkin Elmer Spectrum 2000, Perkin Elmer France, Villebon-sur-Yvette, France) were done before the heat treatment, after the heat treatment and after the wet treatment. Furthermore, determination of the wood constituents transformations obtained were determined by ^13^C NMR and MALDI ToF spectrometry.

### 2.1. Wood Heat Treatment

**Figure 1:**
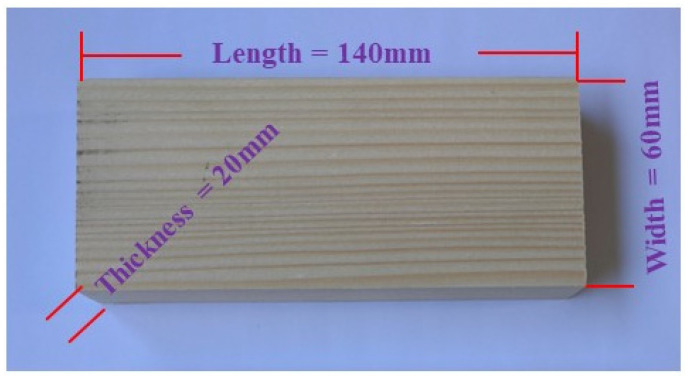
Schematic diagram of wood dimensions for heat treatment.

A hardwood species (beech, *Fagus Sylvatica*) and a softwood species (fir, *Abies alba*) were studied. The wood boards were cut to the dimensions 140 mm (length) × 60 mm(width) × 20 mm(thickness) for heat treatment (Figure 1). Prior to the experiments, the boards were dried in an oven at 103 °C until mass stabilization. The wood specimens obtained under this condition were used as the control group and were called “untreated. Wood heat treatment was realized under different temperatures: 120 °C, 140 °C, 160 °C, 180 °C, 190 °C, 200 °C, 210 °C, 220 °C, 230 °C, 240 °C, 250 °C, 275 °C and 300 °C. The heat treatment was carried out in a reactor placed in a controlled oven under an inert nitrogen atmosphere.

The treatment process was carried out after the drying of the samples until mass stabilization at 103 °C and measurement of the samples weight (*M*1). The heat treatment was done from the room temperature to the target temperature by an increasing of 2 °C/min; the temperature was constant for 120 min and cooled to room temperature without control of the temperature rate of decrease. Afterwards, the weight of heat-treated samples was measured (*M*2), and the mass loss (*ML*) calculated according to Equation (1).

(1) $$ML=\frac{M1-M2}{M1}\times 100\%$$

### 2.2. Wet Treatment

**Figure 2:**
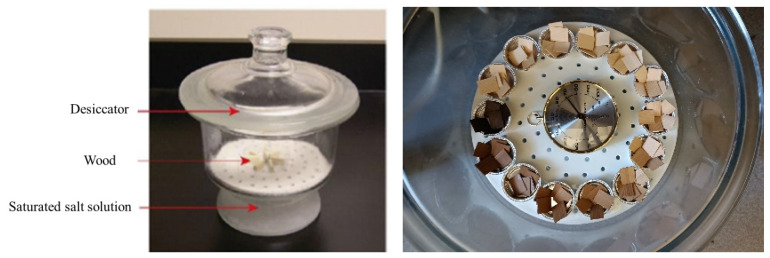
Desiccator used for wood wetting (**left**). Wood samples are placed from low to high heat treatment temperature (**right**).

The wet treatment in this study consisted in placing the specimens after heat treatment in Section 2.1 in a 95% humidity environment for 1 week (7 days) after sawing. Potassium sulfate was used to prepare a suitable amount of saturated solution. This was placed in a closed desiccator (Figure 2), to obtain a relative humidity environment of at least 95%. After the heat-treated wood was placed in an environment at 20 °C at room temperature for 24 h, it was sawed and cut into 24 mm × 20 mm × 1 mm sample slices. A total of 16 of such sample slices were sawn from each temperature-treated board, 8 slices were used to test the advancing contact angle, and the other 8 slices were placed in a 95% relative humidity environment for 7 days. The sheets were removed after 7 days and placed in a desiccator equipped with a desiccant, weighed at determined intervals; then, there was a wait until its quality was consistent with the quality before wet treatment (this process was to ensure that the test pieces for testing the contact angle before and after wet treatment had the same moisture content), and then it was removed to test the advancing contact angle. After the test was completed, the average value of the eight data points was obtained.

### 2.3. Wood Color

Color change of the heat-treated samples was measured by a Chroma Meter CR-410 (KONICA MINOLTA, ZI Paris Nord 2, France) spectrophotometer, according to the CIELab system. Based on the L *, a *, b * color coordinate system, L * represents the black-and-white axis; for black, L * = 0 and for white, L * = 100; a * represents red-green color based on the positive and negative axes and b * represents yellow-blue color (positive value to yellow, negative value to blue). There are two test pieces at each heat treatment temperature. The front and the back sides of the heat-treated samples of each collecting point were photographed. Therefore, the average value of 8 points (two points on each side of each sample) is taken as the final value.

### 2.4. TG/DTG

**Figure 3:**
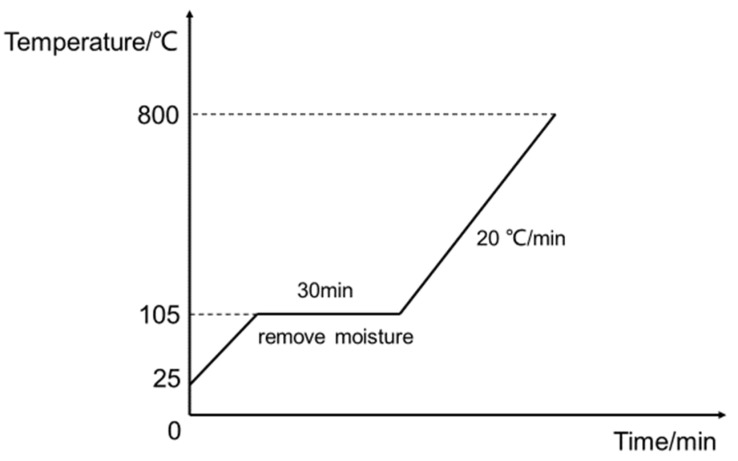
Schematic diagram of temperature rise for TGA/DTG test.

The pyrolysis characteristics of biomass samples were analyzed by a thermogravimetric analyzer (NETZSCH, STA 449 F3 Instruments, Weimar, Germany). In each run, 5–10 mg of sample were loaded into an Al_2_O_3_ crucible, and then the crucible was placed into the instrument TG oven. The N_2_ at a flow rate of 100 mL/min was used as the carrier gas. In TG, the raw or biochar samples were heated from 25 °C at a heating rate of 20 °C/min to 105 °C, followed by keeping this temperature for 30 min to provide a dry-basis sample for the TG. The samples were then heated from 105 °C to 800 °C at the heating rate of 20 °C/min (Figure 3). The tests were followed by thermogravimetric and derivative thermogravimetric (DTG) analyses of untreated and heat-treated beech.

### 2.5. Contact Angle

The advancing contact angles of beech and fir heat-treated at different temperatures were tested by the Wilhelmy method with a Force Tensiometer (KRÜSS France, Villebon-sur-Yvettes, France). Small samples (24 mm × 20 mm × 1 mm) were prepared along the grain from the large specimens (140 mm × 60 mm × 20 mm) before and after heat treatment for testing, and the specimens before and after being heat-treated at each temperature were tested 10 times. The length of each specimen was 24 mm length, 20 mm width, and 1 mm thickness. The immersion depth was 5 mm (in the direction of the width), and the immersion rate was 5 mm/min. The final contact angle (advancing and receding) values are the average of 10 valid tests. Due to heterogeneity and the anisotropic character, it is well known that the value of the receding contact angle with water is always equal to zero on the wood, the dewetting of water is never observed on the wood. In wood after the deposit of a drop of water, it is impossible to remove the water on the surface of the wood, there is no dewetting. This is the reason why, between water and wood, the receding contact angle is always equal to zero.

**Figure 4:**
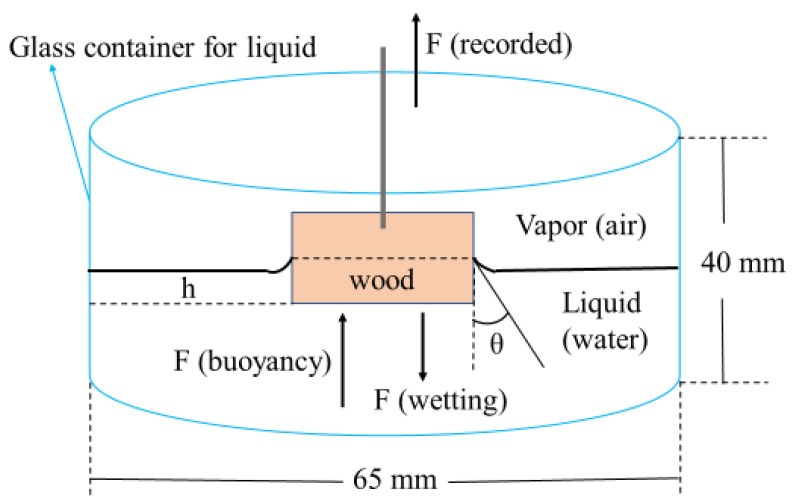
Schematic diagram of the Wilhelmy method.

Figure 4 is a schematic diagram of the Wilhelmy method. Immersing a sample plate of wood into a probe liquid (water) and removing it allows for the determination of advancing and receding contact angles with the Wilhelmy method. In the figure, θ is the contact angle and h is the depth of the wood sample immersed in the liquid (water).

**Figure 5:**
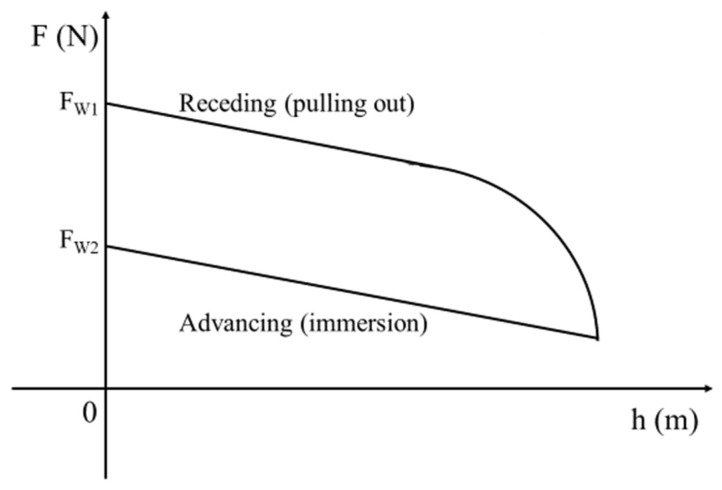
Force recorded by tensiometer as a function of immersion depth.

Figure 5 shows the force recorded by tensiometer as a function of immersion depth. The force on the plate and the immersion depth are measured during the test cycle. The Wilhelmy force F_W_ has an effect only on the liquid surface. This force is constant during the whole immersion and pulling out procedure and is calculated according to equation: F_W_ = cosθ × б × C, where б is the liquid surface tension, C is the wetted circumvented length or perimeter, F_W1_ is the Wilhelmy force of receding, and F_W2_ is the Wilhelmy force of advancing.

### 2.6. FTIR/NIR

The chemical structures of untreated and treated wood samples were analyzed by means of a FTIR/NIR spectrometer (ATR cell on a Perkin Elmer Spectrum 2000, Perkin Elmer France, Villebon-sur-Yvette, France) with attenuated total reflection (ATR). Wood samples were ground to powder with a laboratory knife-mill SM100 (Fritsch, Idar-Oberstein, Germany) equipped with a screen perforated with trapezoidal meshes of 1 mm diameter. The FTIR spectra were recorded in the 4000–650 cm^−1^ range, and 32 scans were collected per run with a spectral resolution of 4 cm^−1^ at room temperature, each sample being tested 10 times.

### 2.7. CP MAS ^13^C NMR

Solid state Cross Polarization-Magic Angle Spinning CP MAS ^13^C NMR was used to characterize the samples. The spectrometer used was an AVANCE II 400 MHz spectrometer (Brüker, Billerica, MA, USA). Furthermore, 100.6 MHz was the frequency used at a sample spin of 12 kHz, and the recycling delay was 1 s, depending on the 1H spin-lattice relaxation times (t1) estimated with the inversion-recovery pulse sequence and a contact time of 1 ms. The decoupling field was 78 kHz, with 15,000 being the number of transients. Tetramethyl silane (TMS) was used as the shift control. The spectra precision was ±1 ppm. Spinning sideband suppression was used. The sample chosen was one at 230 °C to be well in the 200–300 °C range where degradations and rearrangements clearly occur, but at a temperature not so high that oxidation phenomena and other may mask what occurs. The NMR results were interpreted according to established interpretation texts [35,36].

### 2.8. MALDI ToF

Water/acetone (50/50 volume) was used to dissolve the most possible of the samples (4 mg/mL) and the solutions were added to the matrix solution of 10 mg/mL in acetone. 2,5-dihydroxy benzoic acid was used as the matrix to ease placing the sample on the sample-holder plate. Red phosphorus was used for instrument calibration (LaserBio Labs, Valbonne, France)). A concentrated solution of 10 mg/mL of sodium chloride (NaCl) in distilled water was mixed to the matrix to enhance ion formation. The sample was added into the matrix solutions and was divided into three parts of the matrix solution. Three parts of the sample solution and one part of NaCl solution were prepared; 0.5 to 1 µL was put on the MALDI target. The MALDI target was placed into the spectrometer after solvent evaporation. The peaks in the spectrum can present themselves at the actual molecular weight of the chemical species or increased by 23 Da, this being the molecular weight of the Na^+^ linked to the molecule from the NaCl added to the matrix to facilitate the flight of heavier oligomers. Sometimes, both forms of the same species, with and without Na^+^ can be present in the same spectrum. The MALDI-TOF spectra were recorded on an AXIMA Performance instrument (Shimadzu Scientific Instruments, Manchester, UK). The irradiation source was a pulsed nitrogen laser with intervals of 3 ns at a wavelength of 337 nm. The measurements were carried out using the following conditions: polarity: positive; flight path: linear; mass: high (20-kV accelerating voltage) and 100 to 150 pulses per spectrum. The delayed extraction technique was used to apply delay times of 200 to 800 ns, and the ion gate was set at 400 Da. The spectra are exact at ±1 Da.

## 3. Results and Discussion

### 3.1. Mass Loss of Beech and Fir at Different Temperatures

**Figure 6:**
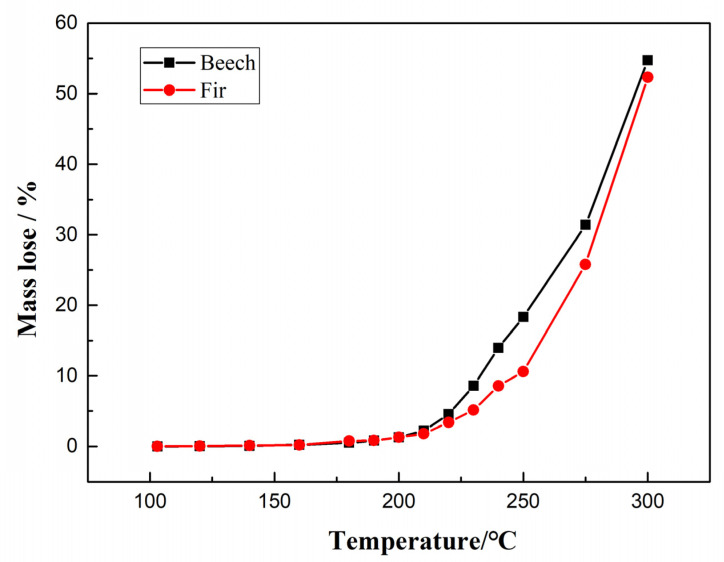
The mass loss of beech and fir at different temperatures.

Figure 6 is a graph of the mass loss trend of beech and fir at different temperatures. It can be seen from the figure that under the same conditions, as the heat treatment temperature increases, the mass loss of the two types of wood shows an upward trend. Between 120–200 °C, the mass loss does not change significantly. Between 200–300 °C, the value of mass loss rises sharply. Compared to beech, the mass loss of fir is slightly lower.

### 3.2. Wood Color (before and after Heat Treatment)

**Figure 7:**
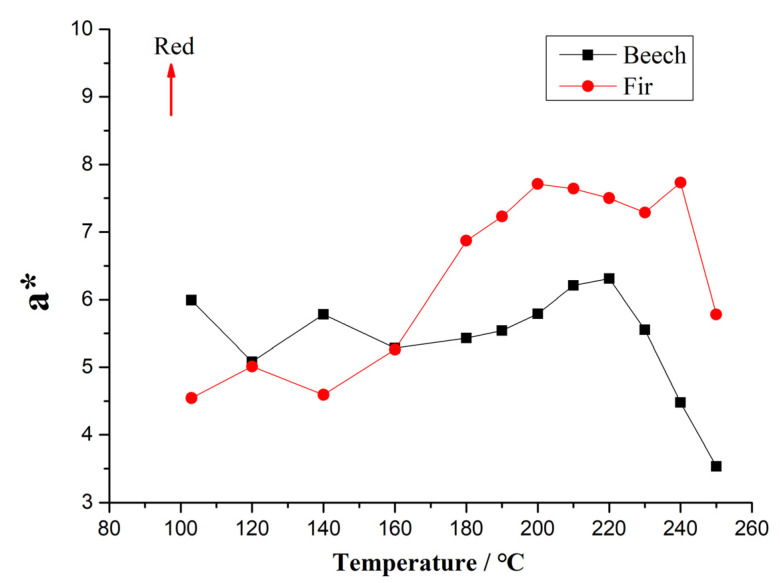

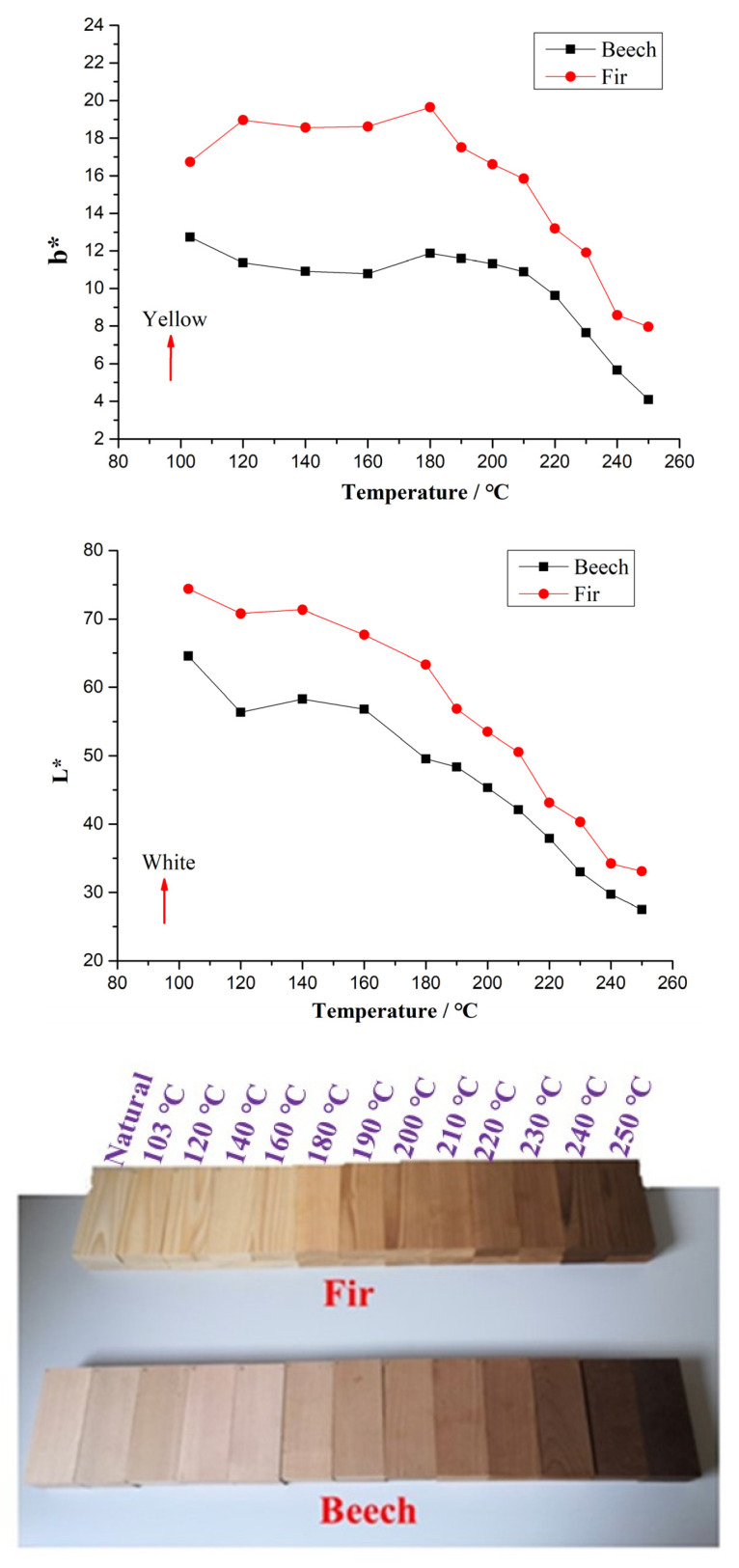
Color changes of color components a*, b* and L* as a function of treatment temperature and visual of color change of beech and fir specimens under treatment temperatures.

Figure 7 shows the color changes of beech and fir specimens at different mass loss values. It can be seen that as the heat treatment temperature continues to increase, the color of the beech and fir specimens will become darker, beech being clearly darker than fir. This is an indication of chemical reactions occurring the reasons of which will become clearer by the chemical analysis. The color of these two wood species before heat treatment is white or yellow. As the heat treatment temperature increases, the degree of white or yellow becomes lower. This change is more obvious in beech than in fir.

### 3.3. TGA/DTG of Beech and Fir at Different Temperatures

**Figure 8:**
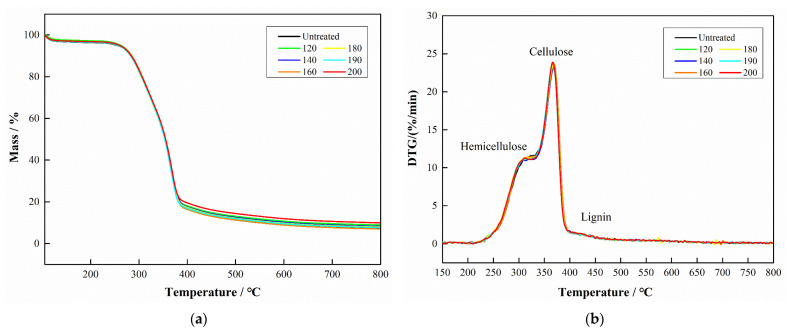
TG curves (**a**) and DTG (**b**) of beech in the heat treatment temperature 120–200 °C range.

For the beech wood, the shoulder temperature (representing hemicelluloses decomposition) on the DTG can be anywhere from 275.1 to 298.2 °C (Figure 8). Most of the hemicelluloses are degraded between 275 and 375 °C at our heating rates [32].

The TG and DTG curves for beech wood pyrolysis at 120–200 °C are shown in Figure 8. They show that in the heat treatment temperature range of 120–200 °C, little or no change in hemicelluloses, cellulose and lignin occurs, and the shape of the curves is similar for all cases.

**Figure 9:**
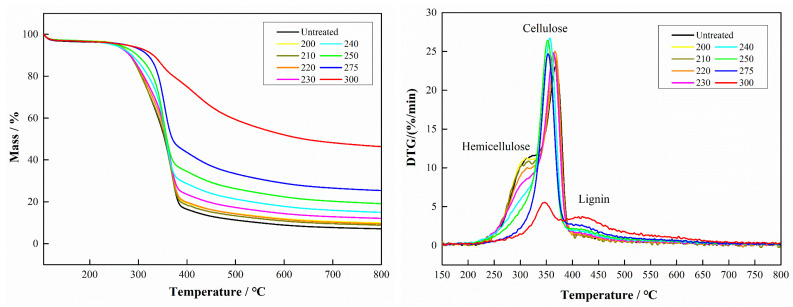
TG curves (**left**) and DTG (**right**) of beech in the heat treatment temperature 200–300 °C range.

The TG and DTG curves for beech wood pyrolysis at 200–300 °C are shown in Figure 9. These show that in the 200–300 °C heat treatment temperature range, changes in hemicelluloses and lignin do occur, while little changes occur in cellulose, and the shapes of the curves are clearly different for all cases. With the increase of heat treatment temperature, the TG curves showed the mass loss is less, indicating that the thermal stability of beech after heat treatment increased proportionally with the increased of the heat treatment temperature. In the DTG curve, with the increase of the heat treatment temperature, the hemicelluloses shoulder gradually decreased and the lignin shoulder gradually increased, indicating that hemicelluloses were the main degraded wood constituent during heat treatment. Consequently, the hydrophilic groups should decrease and the hydrophobicity increase. At the highest temperature of 300 °C, cellulose also showed some degradation.

In the case of fir, a peak at approximately 400 °C appears in the 300 °C heat-treated range, this being mainly lignin, but the shoulder at approximately 375 °C may contain also cellulose. These observations seem to confirm that hemicelluloses degrade in the 200–315 °C range, while cellulose degrades in the 315–400 °C range [33].

**Figure 10:**
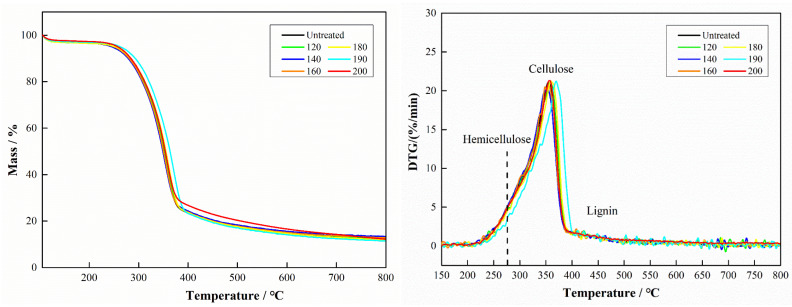
TG curves (**left**) and DTG (**right**) of fir in the heat treatment temperature 120–200 °C range.

**Figure 11:**
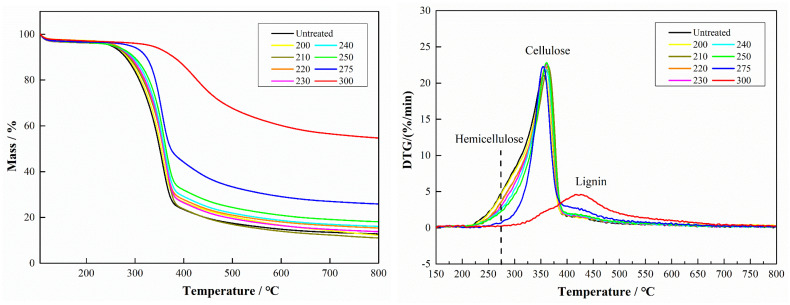
TG curves (**left**) and DTG (**right**) of fir in the heat treatment temperature 200–300 °C range.

The TG and DTG curves for fir wood pyrolysis after heat treatment at 120–200 °C are shown in Figure 10 and in Figure 11 for 200–800 °C. From these two figures, it can be seen that in the heat treatment temperature range of 120–200 °C, as for beech, little or no change in hemicellulose, cellulose and lignin occurs, and the shape of the curves is similar for all cases.

The TG and DTG curves for fir wood pyrolysis at 200–300 °C are shown in Figure 10. They show that in the 200–300 °C heat treatment temperature range, clear changes in hemicelluloses and lignin occur, but little changes in cellulose. The shapes of the curves are clearly different for all cases. The TG curves showed an upward trend with the increase of heat treatment temperature, indicating that the thermal stability of fir after heat treatment improved and was proportional to the increase in heat treatment temperature. The DTG curves showed that as the heat treatment temperature increased, the shoulder of hemicelluloses gradually decreased and lignin gradually increased. This indicates that again hemicelluloses were the main wood constituent degraded during heat treatment. Thus, as for beech, the proportion of hydrophilic groups should decrease, and thus the hydrophobicity should increase. At the highest temperature of 300 °C, cellulose also showed some degradation.

### 3.4. Wettability after Heat-Treatment of Wood and after a Humid Treatment (1 Week at 95% RH)

**Figure 12:**
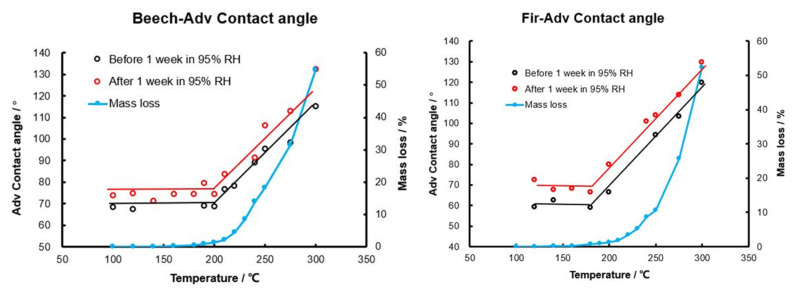
The advancing contact angles of beech (**left**) and fir (**right**) heat-treated at different heat treating temperatures after the humid test by being placed in a 95% relative humidity environment for 1 week.

Figure 12 shows that the contact angle of beech and fir wood before and after heat treatment increased significantly after being placed in a 95% relative humidity environment for one week. When there is no degradation (before 200 °C), there is no change in wettability. For temperatures higher than 200 °C, the degradation of the hemicelluloses starts and the contact angle increases. There is then a significant correlation between degradation temperature of the hemicelluloses and the contact angle. After a 1-week humidity treatment, all of the contact angle values for each temperature treated wood increased. This effect indicated a decrease in the wettability of the heat-treated wood, probably due to a chemical modification of the wood constituents, such as the degradation of hemicellulose, which is consistent with the results of DTG. It means that even after permanence in a very moist environment the decrease in water repellence is rapidly recovered due to the modifications imparted by the heat treatment, contrary to a previous study [7]. Thus, after heat treatment and exposure to a wet atmosphere, the hydrophobic heat-treated timber remains hydrophobic rapidly higher than the original water repellence displayed after the heat treatment. The decrease of water resistance means that when the modified wood is in a humid environment or after raining, some water will enter, resulting in a short-term decrease in the water resistance of the modified wood. However, after the water evaporates (such as after being exposed to the sun), the water resistance will be restored or even improved. This study proves this phenomenon, which is also one of the innovative points of the study. The water wettability of wood is measured under the same conditions of roughness (identical wood machining), moisture content (anhydrous) and fresh surface to highlight the influence of chemical modification. The wetting was therefore tested before and after heat treatment, as well as before and after heat treatment and humid treatment.

### 3.5. FTIR Analysis

**Figure 13:**
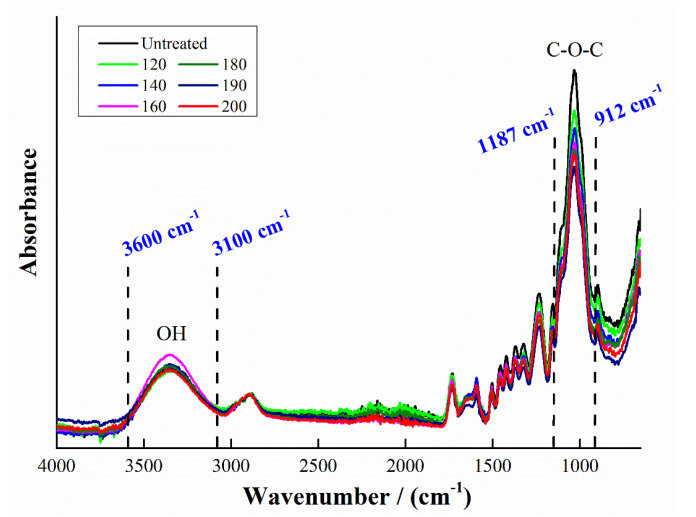
FTIR spectra of beech wood after heat treatment at 120–200 °C.

**Figure 14:**
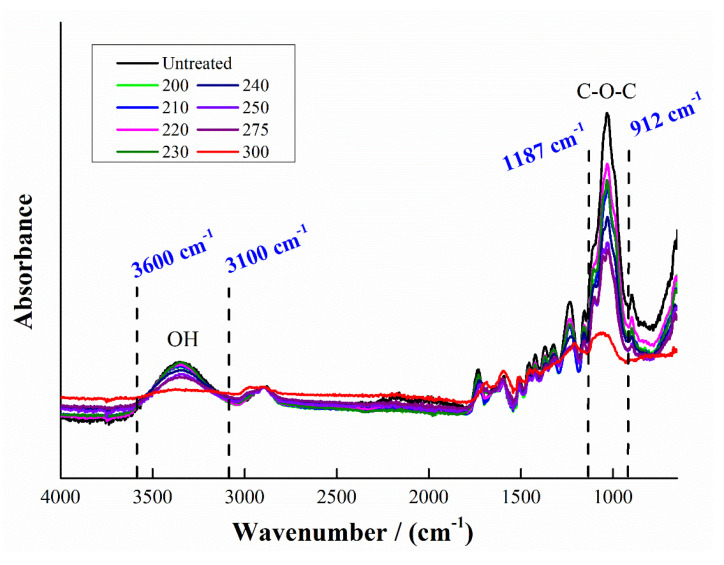
FTIR spectra of beech wood after heat treatment at 200–300 °C.

Figure 13 and Figure 14 are the infrared spectra of beech wood after heat treatment at different temperatures. It can be seen from the figure that the functional groups related to wood wettability are mainly -OH and C-O-C groups; these two functional groups are mainly reflected in the wavenumber range of 3600–3100 cm^−1^ and 1187–912 cm^−1^.

These are in line with previous studies by where FTIR changes in the chemical structure of the hemicelluloses, cellulose, and lignin of Chinese fir wood were observed from the FT-IR spectra [30], namely: the degradation of some pyranose rings in hemicelluloses, a decrease in cellulose crystallinity, and a loss of C=O and C=C groups in the aromatic skeleton. These authors [30] also did 2D-IR spectra with the results indicating hemicellulose degradation. The changes at 1627 and 1509 cm^−1^ at high temperature also indicated cross-linking by rearrangement of the lignin aromatic moieties.

Another study [26] on a type of fir and on hornbeam woods indicated a reduction of the number of -OH groups after a high temperature treatment, and etherification occurring as shown by the increase in a specific carbonyl peak above 150 °C in the heat-treated wood FTIR spectrum. However, cross-linking had a much more marked effect than a very modest effect due to etherification reactions in decreasing swelling.

The FTIR spectra in Figure 13 and Figure 14 also show a progressive decrease in -OH groups in the 200–300 °C range. In order to more clearly understand the wettability of beech and fir wood after heat treatment at different temperatures, the curves corresponding to -OH groups were enlarged for analysis. These are shown in Figure S1 (Supplementary Materials). Similar trends are also observed for fir. Figure S1 (Supplementary Materials) shows the -OH areas, respectively, in FTIR spectra of beech wood after heat treatment at different temperatures. It can be seen from them that below 200 °C, the difference in the absorption peak intensity of -OH is not obvious. Above 200 °C, the intensity of the -OH absorption peak was significantly lower than that of the wood before heat treatment, indicating that the wood heat-treated above 200 °C was less likely to absorb moisture. The same trends are also present for the FTIR spectra of fir wood. Additionally, in Figure 14 and its magnification (Figure S2, Supplementary Materials), the C-O-C bands at 1187–912 cm^−1^ centered at 1040 cm^−1^ decrease little up to 200 °C but decrease sharply and progressively with the increase of temperature above 200 °C, these bands being characteristic of polymeric carbohydrates. This indicates degradation of hemicelluloses by progressive cleavage of some glucosidic bridges as well as pyranose ring opening. While all of this is in accord with previous work, it is also clear that FTIR analysis alone is not sufficient to explain the reactions occurring, the products formed, and even less able to explain the progressive hydrophobicity as a function of the increasing heat treatment temperature.

### 3.6. CP MAS ^13^C NMR

**Scheme 1:**
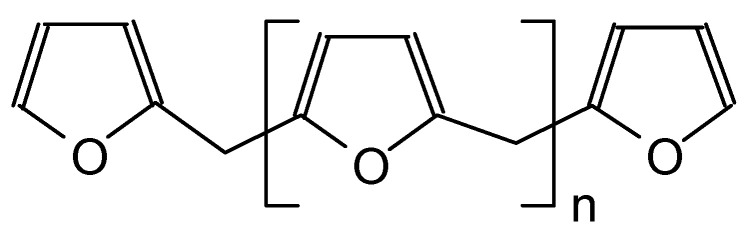
Type I.

**Scheme 2:**
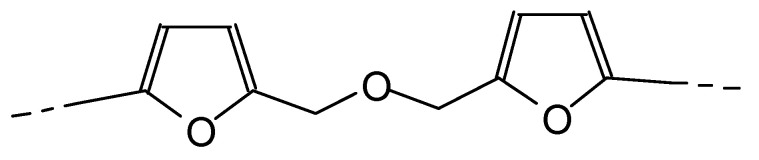
Type II.

Of particular interest in the CP MAS 13C NMR of beech heat-treated at 230 °C is the presence of pronounced peaks at 149 and at 148 ppm, which are absent or almost completely absent in an untreated beech wood control. Of these, the 149 ppm peak may corresponds to an aromatic carbon bonded to a non-aromatic carbon, thus ArC-C. This may be either a type of bond other than the normal bonds present in lignin or of the same type but in considerable excess than what is usually found, indicating rearrangements of lignin. However, it is also characteristic of furanic resins in which the C2 and C5 positions are substituted, thus of furanic resins where the furanic moieties are linked both by their C2 and their C5 to other furanic moieties in the middle of linear furanic resins with linkages of type I and II (Scheme 1 and Scheme 2).

And also

**Figure 15:**
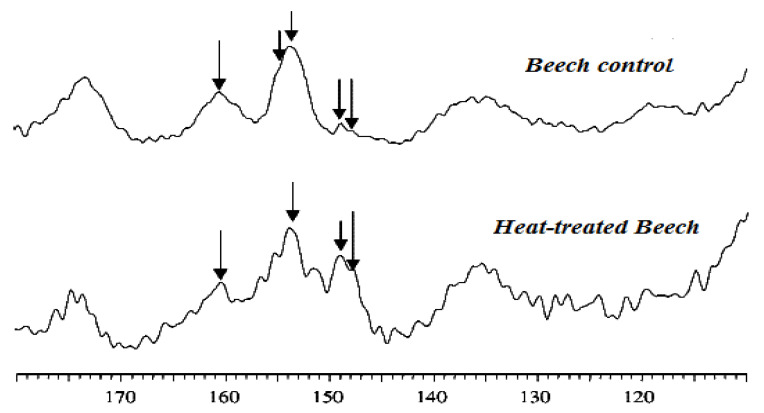
CP MAS ^13^C NMR spectra of untreated beech wood and beech wood heat-treated at 230 °C in the 110–180 ppm.

This infers that a reaction introducing a substituent on the aromatic ring of lignin has occurred or that furanic resins have been formed. That this is the case is also shown by the greater number of peaks present in the crowded 147–165 ppm range in the heat-treated wood in relation to the beech control. However, the most interesting peak is at 148 ppm, which is characteristic of the carbons of a furanic ring [35,36], and in particular of the free C5 site of furfural [35,37]. Furthermore the signals of furanic rings C2 and C5 linked by a methylene bridge should appear at 155 ppm indicating that furanic resins of some type have formed as well. Furfural, hydroxyl methyl furfural and other furanic materials are well known to be generated from wood carbohydrates [38,39,40,41,42,43], here quite likely being catalyzed from the acetic acid generated by the heat treatment induced hydrolysis of the acetyl groups on hemicelluloses [44], this being a well-known and studied reaction. The high temperatures between 200–300 °C used for the heat treatment, where other techniques have already shown reactions to occur (Figure 15), indicates that formation of furfural in the heat-treated wood may well occur and that this polymerizes to structures of the type.

This type of structures will also account for the 149 ppm peak, confirming the presence and formation of furanic resins. The presence of the smaller peaks at 152 ppm and 161–162 ppm also indicate that hydroxymethyl furfural has formed, although in lesser proportions. The fact is that the 148 ppm peak is more marked in heat-treated wood samples; the higher treating temperature indicates that this is indeed the peak of the C5 of a furan ring. A peak of the same order of size should however appear in the 152–155 ppm range for the furan C2 [35,36,37], as indeed it does. A number of peaks fulfilling this requirement are indeed present although the most likely candidate peak is slightly more marked as it is likely to be a superposition of the furan C2 with another signal. The carboxylates band at 173 ppm decreases in heat-treated wood, indicating the heat induced hydrolysis of the acetyl groups on the hemicelluloses [43], the acetic acid so formed renders sufficiently acid the environment to catalyze the formation of furanic moieties [38,39,40,41,42,43]. In all cases, all this indicates is an increase in cross-linking of the system either due to rearrangement of the lignin with itself or of the lignin with other wood constituents, namely, furanic resins generated from the carbohydrates, thus an increase in the water repellence of the heat-treated wood. The darkening of the wood with the increasing treating temperature is due not only to oxidation reactions but mainly by the increase in the proportion of the black furanic resins generated by the degradation of the hemicelluloses.

### 3.7. MALDI ToF

**Scheme 3:**
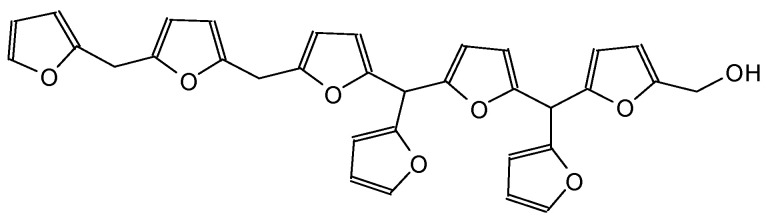
III.

**Figure 16:**
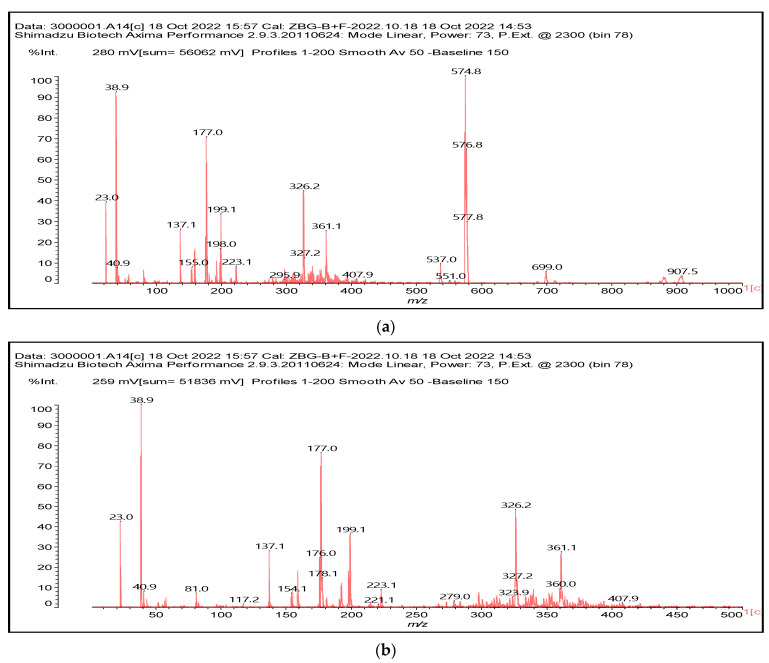

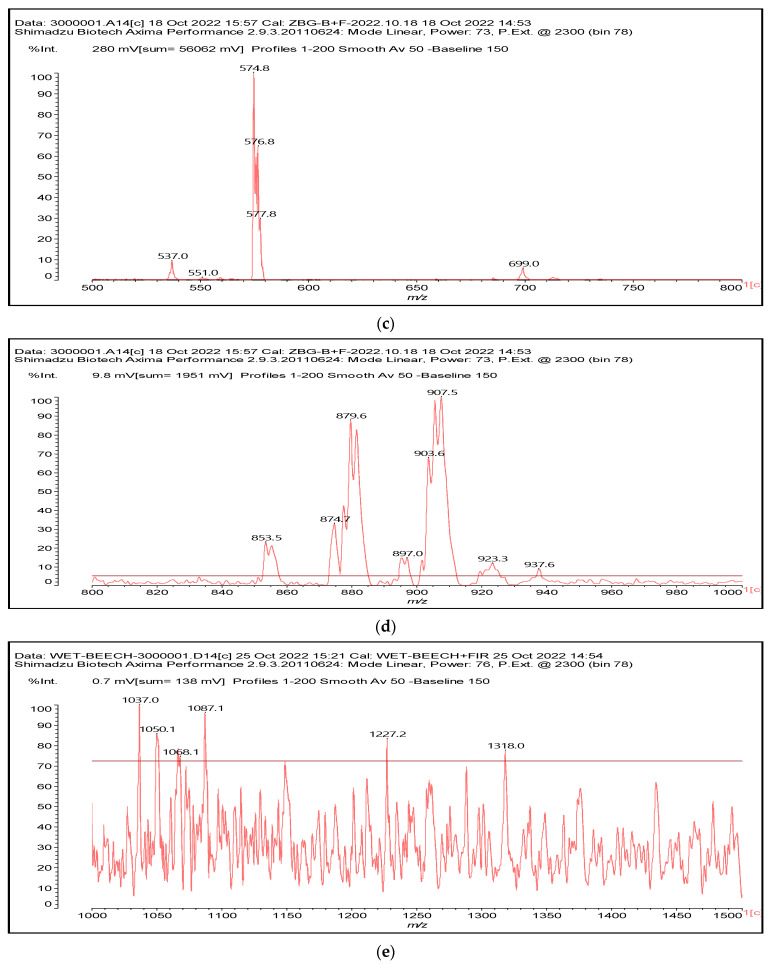
MALDI ToF spectra of heat-treated beech at 300 °C in the (**a**) 20–1000 Da range and details of the (**b**) 20–500 Da range, (**c**) 500–800 Da range, (**d**) 800–1000 Da range, (**e**) 1000–1500 Da range.

The MALDI ToF spectra of the 300 °C heat-treated beech wood (Figure 16a–e) show a great majority of furanics oligomers of higher molecular weight. One can notice in the first general spectrum (Figure 16a) at 300 °C the predominance of the peak at 574 Da (III in Scheme 3), thus a branched oligomer showing the start of cross-linking in relation instead to the predominance of the smaller molecular weight species at 200 °C.

**Scheme 4:**
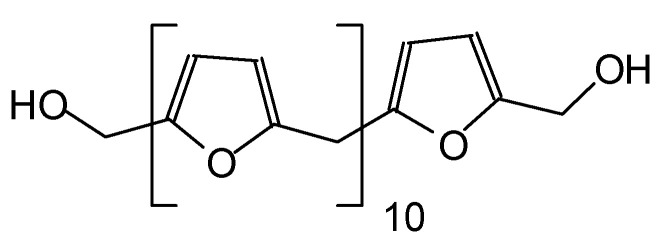
IV.

It is interesting with the 907 Da peak being higher in the general spectrum at 200 °C than at 300 °C, that the predominant higher molecular weight oligomers at 200 °C are linear (Figure S3, Table S1, Supplementary Materials) and not branched as at 300 °C. The same trend is valid at 103 °C but with the low molecular weight species greatly predominating and also some linear oligomers (Figure S4, Supplementary Materials) but no branched species thus with no cross-linking, but the linear oligomers present showing that polymerization already starts to occur at this lower temperature. In Table 1 are shown all the species assigned to the peaks in the spectra in Figure 16a–e. In Table 1 are also underlined the species of the peaks that are present in the MALDI spectra after the wet treatment of the beech timber. The majority of the same water repellant furanic species are still present after the wet treatment explaining at the molecular level why the water repellence of the heat-treated timber is conserved and increased after a wet treatment. Branched species, thus the precursors of the start of the system cross-linking, such as those at 551 Da, 574 Da, 699 Da, 1037 Da, 1068 Da, 1151 Da and 1227 Da are present. Many lower molecular weight species as well as linear higher molecular weight oligomers are also present, such as the species at 324 Da, 361 Da, 853 Da, 882 Da, 897 Da, 904 Da, 937 Da (IV in Scheme 4) and 942 Da.

The same species are also formed in the heat treatment of fir wood (Figures S5 and S6, Supplementary Materials). At 300 °C fir wood is strongly and mostly cross-linked with the cluster at approximately 574 Da totally predominating (Figure S5). The lower molecular weights and linear oligomers are in great minority (only the 177 Da is present and is very small) and all the others are absent. There are some fragments of linear oligomers in the cluster at approximately 324 Da (one can see the 324 Da no Na^+^, and the 347 Da with Na^+^), but that is all. The furanics are more cross-linked than in beech.

At 103 °C the small linear species predominate and there is some start of cross-linking (the 537 Da peak) but at the smaller molecular weights. It means that the furanic oligomers have formed but at smaller molecular weight and are predominantly linear (Figure S6).

In the case of fir wood, the trends are the same but with some differences (Figures S5 and S6, Supplementary Materials). The species observed are strongly present and mostly cross-linked with the cluster at approximately 574 Da totally predominating at 300 °C. The lower molecular weights and linear oligomers are in great minority (only the 177 Da is present and very small) and all the others are absent. There are some fragments of linear oligomers in the cluster at approximately 324 Da (one can see the 324 no Na^+^, and the 347 Da with Na^+^), but that is all. The furanic oligomers are more branched/cross-linked than in beech wood at 300 °C.

## 4. Conclusions

The work presented here addresses three points of interest in hydrophobation by heat treating wood. First of all, contrary to a previous study, after heat treatment and exposure to a wet atmosphere the hydrophobic heat-treated timber remains hydrophobic rapidly regaining the original water repellence displayed after the heat treatment. At the same temperature of the previous study at 120 °C, the temperature is too low to form furanics in any great amounts to show significant differences. The water wettability of wood is measured under the same conditions of roughness (identical wood machining), moisture content (anhydrous) and fresh surface to highlight the influence of chemical modification. The wetting was therefore tested before and after heat treatment. The wood becomes hydrophobic and this phenomenon is attributed to the chemical modification of the wood. The wood becomes hydrophobic with more thermal degradation and increases in the contact angle. We wanted to see if after a heat treatment and after a wet treatment, the hydrophobicity generated by the thermodegradation was preserved. It is showed that after wet exposure, there is no notable modification of the contact angle. A slight increase of the contact angle is measured. This phenomenon is most likely due to aging (1 week) of the surface during wet processing. It can also be noted that exposure to the humid atmosphere does not change the wettability of heat-treated wood. After a heat treatment and a wet treatment, the wood becomes hydrophobic and this property remains constant after a humid exposure. Second, while the FTIR analysis confirmed the previous rather limited insight in the chemical transformations induced by heat treatment, analysis by CP MAS ^13^C NMR and MALDI ToF mass spectrometry has allowed for defining the reactions leading to wood hydrophobation. The indications obtained were that wood hydrophobation is caused by the formation of furanic compounds, mainly furfural, but also in minor proportions of hydroxymethyl furfural derived from the hemicelluloses degradation catalyzed by the acetic acid generated by the hydrolysis of their acetyl groups. The furanic compounds formed then both progressively polymerize to strongly water repellant, insoluble furanic resins finally forming a water repellant network throughout the heat-treated wood. The concomitant progressive darkening of the wood as a function of the increasing heat treatment temperature is not only due to some oxidation of wood constituents but mainly by the increasing proportions of the tri-dimensionally cross-linked black-colored, water repellant furanic resins network throughout the wood. It is by far and mainly an interpenetrating furanic network, although reaction with lignin moieties cannot be excluded. In fact, the furanic compounds that are generated from the hemicelluloses can react with lignin sites as well, but this is not possible to analyze as the experiments were carried out on solid wood samples, hence with lignin fixed in its wood network. This mechanism is particularly evident in the 200 to 300 °C temperature range. This study also proves that heat-treated wood will still have good water resistance after experiencing harsh environments, such as rain.

It is true that in in other laboratories, mass losses can be observed for lower treatment temperatures (see references in the introduction). Below 170 °C it is always surprising to observe a loss of mass. This observation may correspond to the evaporation of gum and resin (free extracts). Between 170 and 200 °C, some authors observe a loss of mass, which is not the case in our experiment. This can be explained by the following factors:

- Wood species and their chemical compositions do not behave in the same way;
- The temperature increase speeds are different;
- Under which type of atmosphere (nitrogen in our case) the experiments are conducted. Under a humid atmosphere we can observe a thermohydrolysis phenomenon which starts at a lower temperature, and under a slightly oxidizing atmosphere (presence of oxygen in the smoke) we can observe a slight phenomenon of combustion before 200 °C;
- The size of the sample has a bearing. Between powder samples as in previous literature, and solid wood as used in the present experiments, the thermal transfer does not take place in the same manner, thus the thermal energy is not transferred in the same manner (the degradation is activated by heating);
- The conductive or convective heat transfer also modifies the heat transfer to the wood.

For these reasons, it is normal to observe the beginnings of thermal degradation under pyrolysis conditions for different temperatures. All of the studies were not carried out under the same conditions of wood species, rate of temperature increase, atmosphere conditions, sample size, and the nature of heat transfer. Finally, it is always difficult to compare industrial studies with laboratory studies.

## Figures and Tables

**Table 1:** Assignment of species to the MALDI ToF peaks for beech wood heat-treated at 300 °C after heat treatment and underlined in green after a high moisture treatment at 95% relative humidity for one week.

Peaks with Asterisks (**) Are also Present after the One Week Wet Treatment
117 Da ** = Furfural, with Na^+^, deprotonated (calculated 118 Da)
177 Da ** = no Na^+^, with 176 deprotonated, 178 normal (Calc 178 Da).The second one is 176 Da exactly
199 Da ** = 177 Da with Na^+^
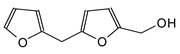 **Or** 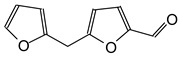 **Or** 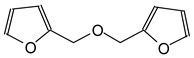
279 Da = with Na^+^, deprotonated calculated 279 Da
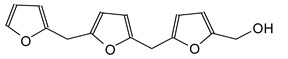
324–326 Da ** = no Na^+^, protonated, calc.324–325 Da
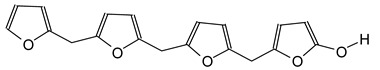
**361 Da** ** = with Na^+^ (calculated 361 Da)
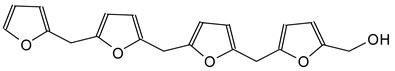
537 Da ** = with Na^+^ Calculated 537 Da
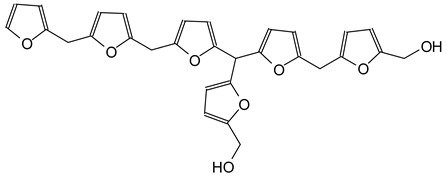
551 Da = no Na^+^
574 Da ** = with Na^+^
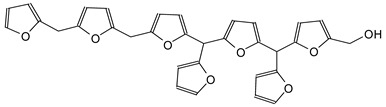
699 Da ** = with Na^+^, deprotonated, calculated 700 Da
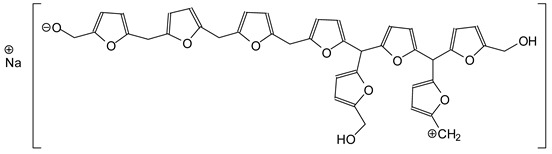
853 Da ** = with Na^+^, calculated 849 D
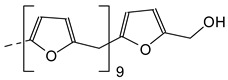
882 Da ** = with Na^+^, deprotonated, calculated 882 Da, **only in WET**
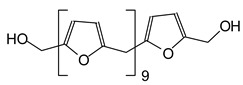
897 Da ** = with Na^+^, calculated 897 Da, **only in WET**
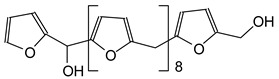
904–907 Da ** = no Na^+^, calculated 905 Da, one specie deprotonated, one species protonated
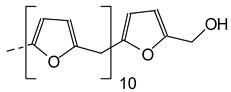
937 Da ** = no Na^+^, calculated 937 Da
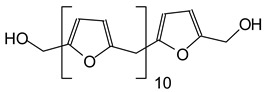
942 Da ** = with Na^+^,
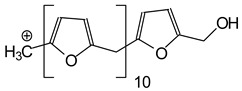
1037 Da ** = with Na^+^, deprotonated Calculated 1037 Da
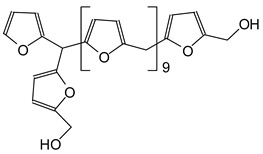
1068 Da ** = with Na^+^, deprotonated
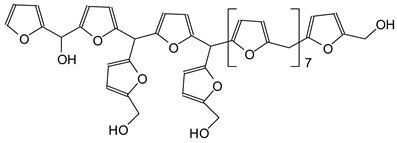
1151 Da ** = with Na^+^
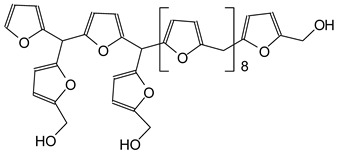
1227 Da ** = with Na^+^
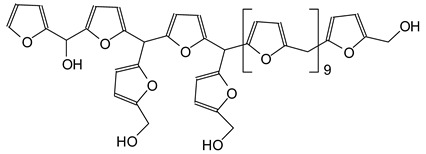

## Data Availability

All data are only availablein the publication and in the supplementary material.

## Supplementary Materials

The following supporting information can be downloaded at: https://www.mdpi.com/article/10.3390/polym15010221/s1, Figure S1: Magnification of -OH FTIR spectra range of beech wood after heat treatment in the 120–200 °C range (**left**) and in the 200–300 °C range (**right**); Table S1: Assignment of species to the MALDI ToF peaks for beech wood heat-treated at 200 °C after heat treatment; Figure S2: C-O-C FTIR spectra of beech wood after heat treatment in the 120–200 °C range (**left**) and in the 200–300 °C range; Figure S3: MALDI ToF spectra of heat-treated beech at 200 °C in the (**a**) 20–1000 Da range and details of the (**b**) 20–500 Da range, (**c**) 500–800 Da range, (**d**) 800 1000 Da range, (**e**) 1000 -1500 Da range (**right**); Figure S4: MALDI ToF spectra of heat-treated beech at 103 °C in the (**a**) 20–1200 Da range and details of the (**b**) 20–500 Da range; Figure S5: MALDI ToF spectra of heat-treated fir wood at 300 °C in the (**a**) 20–1000 Da range and in the (**b**) 20–500 Da range; Figure S6: MALDI ToF spectra of heat-treated fir wood at 103 °C in the (**a**) 20–1000 Da range.

## References

[1] Gardner D.J., Generalla N.C., Gunnells D.W., Wolcott M. (1991). Dynamic wettability of wood. Langmuir.

[2] Mantanis G.I., Young R.A. (1997). Wetting of wood. Wood Sci. Technol..

[3] Pétrissans M., Gérardin P., Bakali I.E., Serraji M. (2003). Wettability of heat-treated wood. Holzforschung.

[4] Hakkou M., Pétrissans M., Zoulalian A., Gerardin P. (2005). Investigation of wood wettability changes during heat treatment on the basis of chemical analysis. Polym. Degrad. Stabil..

[5] Gérardin P., Petrič M., Petrissans M., Lambert J., Ehrhrardt J.J. (2007). Evolution of wood surface free energy after heat treatment. Polym. Degrad. Stabil..

[6] Petrič M., Knehtl B., Krause A., Militz H., Pavlic M., Petrissans M., Rapp A., Tomazic M., Welzbacher C., Gerardin P. (2007). Wettability of waterborne coatings on chemically and thermally modified pine wood. J. Coatings Technol. Res..

[7] Endo K., Obataya E., Zeniya N., Matsuo M. (2016). Effects of heating humidity on the physical properties of hydrothermally treated spruce wood. Wood Sci. Technol..

[8] Bekhta P., Niemz P. (2003). Effect of high temperature on the change in color, dimensional stability and mechanical properties of spruce wood. Holzforschung.

[9] Sivonen H., Maunu S.L., Sundholm F., Jämsä S., Viitaniemi P. (2002). Magnetic resonance studies of thermally modified wood. Holzforschung.

[10] Bourgeois J., Janin G., Guyonnet R. (1991). The color measurement: A fast method to study and to optimize the chemical transformations undergone in the thermically treated wood. Holzforschung.

[11] Windeisen E., Strobel C., Wegener G. (2007). Chemical changes during the pro-duction of thermo-treated beech wood. Wood Sci. Technol..

[12] Windeisen E., Bächle H., Zimmer B., Wegener G. (2009). Relations between chemical changes and mechanical properties of thermally treated wood 10th EWLP, Stockholm, Sweden, 25–28 August 2008. Holzforschung.

[13] Wålinder M.E.P., Johansson I. (2001). Measurement of wood wettability by the Wilhelmy method. Part 1. Contamination of probe liquids by extractives. Holzforschung.

[14] Wålinder ME P., Ström G. (2001). Measurement of wood wettability by the Wilhelmy method. Part 2. Determination of apparent contact angles. Holzforschung.

[15] Moghaddam M.S., Walinder M.E.P., Claesson P.M., Swerin A. (2013). Multicycle Wilhelmy plate method for wetting properties, swelling and liquid sorption of wood. Langmuir.

[16] Moghaddam M.S., Walinder M.E.P., Claesson P.M., Swerin A. (2016). Wettability and swelling of acetylated and furfurylated wood analyzed by multicycle Wilhelmy plate method. Holzforschung.

[17] Chien Y.-C., Yang T.-C., Hung K.-C., Li C.-C., Xu J.-W., Wu J.-H. (2018). Effects of heat treatment on the chemical compositions and thermal decomposition kinetics of Japanese cedar and Beech wood. Polym. Degrad. Stabil..

[18] Ozgenc S.D.O., Boyaki I.A., Eksi-Kocak H. (2018). ATR-FTIR Spectroscopic Analysis of Thermally Modified Wood Degraded by Rot Fungi. Drewno.

[19] Hoseinzadeh F., Zabinzadeh S.M., Dastoorian F. (2019). Creep behavior of heat-treated Beech wood and the relation to its chemical structure. Constr. Build. Mater..

[20] Zhou Y., Zuo Y., Wu Y., Yuan G., Li X. (2020). Construction of a network structure in Chinese Fir wood by Na_2_SiF_6_ crosslinked Na_2_SiO_3_. J. Mater. Res. Technol..

[21] Ali M. (2021). Weathering of Outdoor Beech Wood and Methods of Conservation. Conserv. Sci. Cult. Heritage..

[22] Hofmann T., Tolvaj L., Visi-Rajczi E., Varga D. (2022). Chemical changes of steamed timber during short-term photodegradation monitored by FTIR spectroscopy. Eur. J. Wood Prod..

[23] Jakob M., Czabany I., Veigel S., Muller U., Gindl-Altmutter W. (2022). Comparing the suitability of domestic spruce, beech, and poplar wood for high-strength densified wood. Eur. J. Wood Prod..

[24] Tjeerdsma B.F., Militz H. (2005). Chemical changes in hydrothermal treated wood: FTIR analysis of combined hydrothermal and dry heat-treated wood. Holz Roh Werkst..

[25] Nguila-Inari G., Petrissans M., Lambert J., Erhardt J.J., Gerardin P. (2006). XPS characterization of wood chemical composition after heat-treatment. Surface Interface Anal..

[26] Aydemir D., Gunduz G., Altuntas E., Ertas M., Turgut S.H., Alma M.H. (2011). Investigating Changes in the Chemical Constituents and Dimensional Stability of Heat-Treated Hornbeam and Uludag Fir Wood. Bioresourses.

[27] Huang X., Kocaefe D., Kocaefe Y., Boluk Y., Pichette A. (2012). Changes in wettability of heat-treated wood due to artificial weathering. Wood Sci. Technol..

[28] Esteves B., Velez Marques A., Domingos I., Pereira H. (2013). Chemical Changes of Heat-treated Pine and Eucalypt Wood Monitored by FTIR. Maderas Cienc. Tecnol..

[29] Yildiz S., Tomak E.D., Yildiz U.C., Ustaomer D. (2013). Effect of artificial weathering on the properties of heat-treated wood. Polym. Degrad. Stabil..

[30] Cheng S., Huang A., Wang S., Zhang Q. (2016). Effect of Different Heat Treatment Temperatures on the Chemical Composition and Structure of Chinese Fir Wood. Bioresourses.

[31] Özgenç O., Durmaz S., Boyaci I.H., Eksi-Kocak H. (2017). Determination of chemical changes in heat-treated wood using ATR-FTIR and FT Raman spectrometry. Mol. Biomol. Spectr..

[32] Anca-Couce A., Tsekos C., Retschitzegger S., Zimbardi F., Funke A., Banks S., Kienzl N. (2020). Biomass pyrolysis TGA assessment with an international round robin. Fuel.

[33] Lin B.J., Colin B., Chen W.H., Pétrissans A., Rousset P., Pétrissans M. (2018). Thermal degradation and compositional changes of wood treated in a semi-industrial scale reactor in vacuum. J. Anal. Appl. Pyrolysis.

[34] Tian M., Zhang B., Wu Z., Yu L., Li L., Xi X. (2021). Effects of Steam Heat-Treatment on Properties of *Pinus massoniana* Wood and Its Bonding Performance. J. Renew. Mater..

[35] Pretsch E., Clerc T., Seibl J., Simon W. (1989). Tables of Spectral Data for Structure Determination of Organic Compounds: ^13^C-NMR, 1H-NMR, IR, MS, UV/VIS.

[36] Wehrli F.W., Wirthlin T. (1978). Interpretation of ^13^C NMR Spectra.

[37] Gfeller B., Zanetti M., Properzi M., Pizzi A., Pichelin F., Lehmann M., Delmotte L. (2003). Wood bonding by vibrational welding. J. Adhesion Sci. Technol..

[38] Fengel D., Wegener G. (1989). Wood: Chemistry, Ultrastructure, Reactions.

[39] van Zandvoort I., Wang Y., Rasrendra C.B., van Eck E.R.H., Bruijnincx P.C.A., Heeres H.J., Weckhuysen B.M. (2013). Formation, Molecular Structure, and Morphology of Humins in Biomass Conversion: Influence of Feedstock and Processing Conditions. ChemSusChem.

[40] van Zandvoort I., Koers E.J., Weingarth M., Bruijnincx P.C.A., Baldus M., Weckhuysen B.M. (2015). Structural characterization of ^13^C-enriched humins and alkali-treated ^13^C humins by 2D solid-state NMR. Green Chem..

[41] Chen X., Guigo N., Pizzi A., Sbirrazzuoli N., Li B., Fredon E., Gerardin C. (2020). Ambient Temperature Self-Blowing Tannin-Humins Biofoams. Polymers.

[42] Sun S., Zhao Z., Umemura K. (2019). Further Exploration of Sucrose-Citric Acid Adhesive: Synthesis and Application on Plywood. Polymers.

[43] Zhao Z., Sakai S., Wu D., Chen Z., Zhu N., Huang C., Sun S., Zhang M., Umemura K., Yong Q. (2019). Further Exploration of Sucrose-Citric Acid Adhesive 2: Investigation of Optimal Hot Pressing Conditions for Plywood and Curing Behavior. Polymers.

[44] Tjeerdsma B.F., Boonstra M., Pizzi A., Tekely P., Militz H. (1998). Characterization of thermally modified wood: Molecular reasons for wood performance improvement. Holz Roh Werkst..

