# Supplementary material for: Furanic Polymerization Causes the Change, Conservation and Recovery of Thermally-Treated Wood Hydrophobicity before and after Moist Conditions Exposure
Source: Polymers (Basel). 2022 Dec 31;15(1):221. doi: 10.3390/polym15010221 (PMC9823973; doi:10.3390/polym15010221)
Supplement: Supplementary file 1 [file polymers-15-00221-s001.zip › polymers-2089913-supplementary.pdf]

Supplementary Material

Chemical Causes of the Change, Conservation and Recovery of Thermally Treated Wood Hydrophobicity Before and After Moist Conditions Exposure.

Bengang Zhang, Matthieu Petrissans*, Anneli Petrissans, Antonio Pizzi*

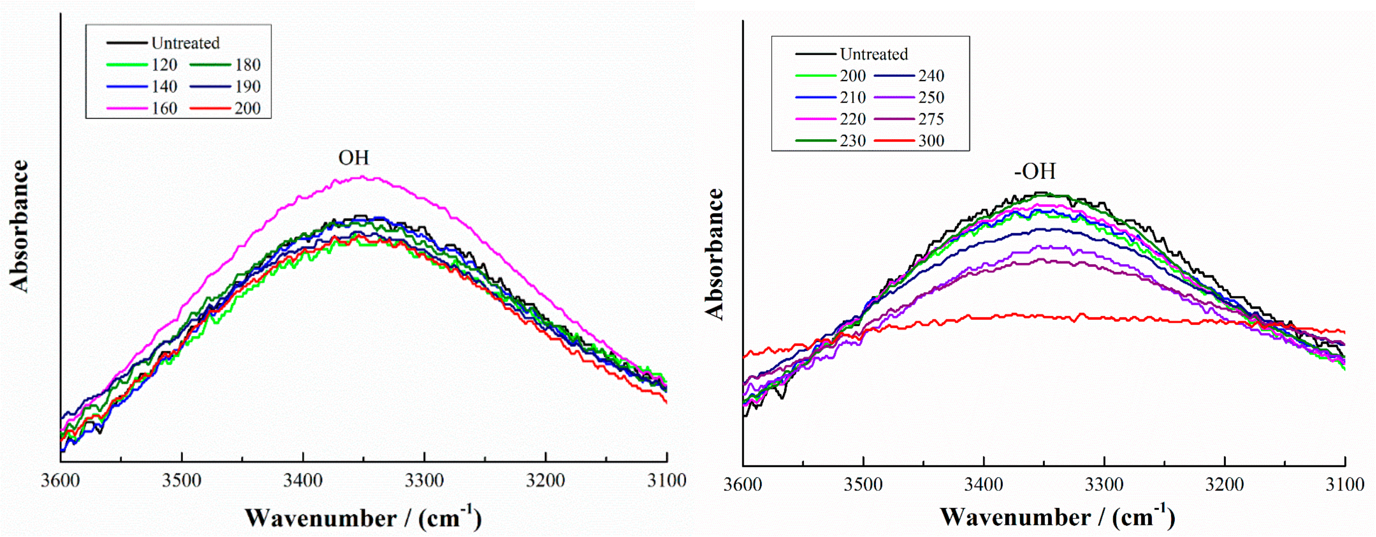

Figure S1. Magnification of -OH FTIR spectra range of Beech wood after heat treatment in the 120-200°C range (left), and in the 200°C-300°C range (right)

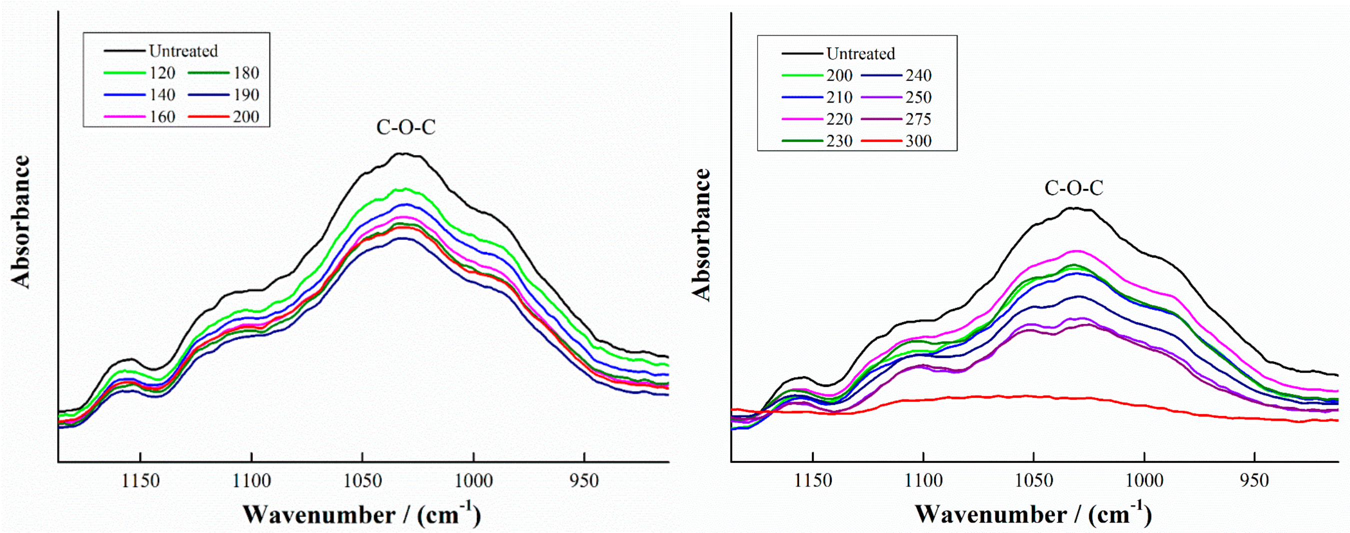

Figure S2. C-O-C FTIR spectra of Beech wood after heat treatment in the 120-200°C range (left) and in te 200-300°C range (right)

MALDI ToF SPECTRA OF BEECH WOOD TREATED AT 200°C

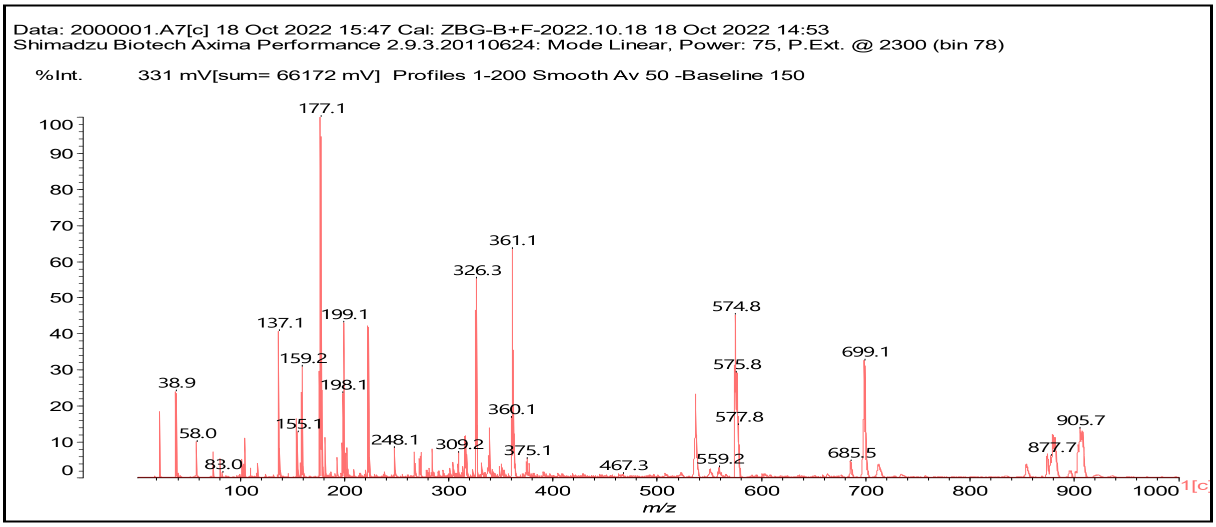

(a)

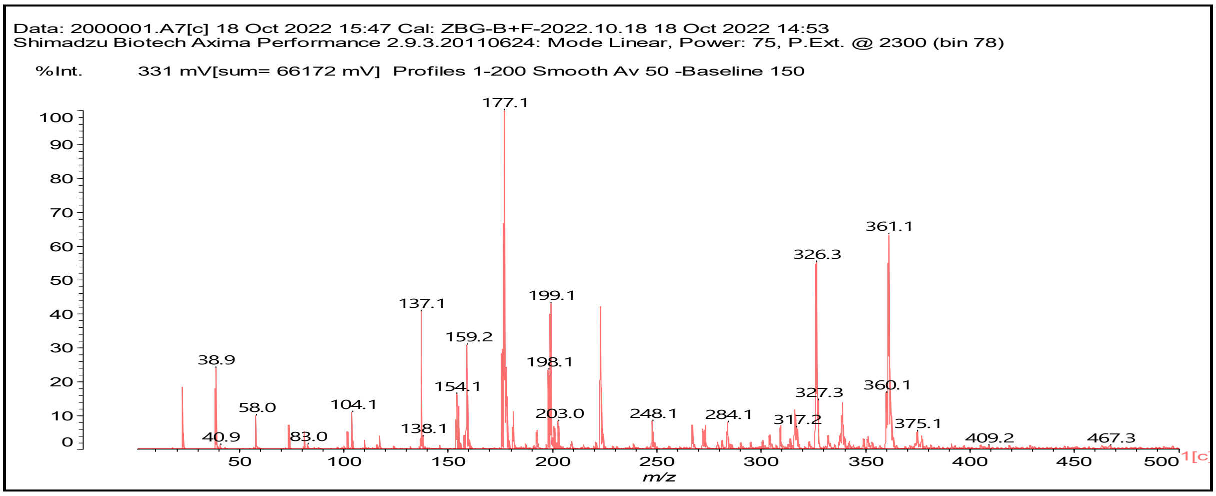

(b)

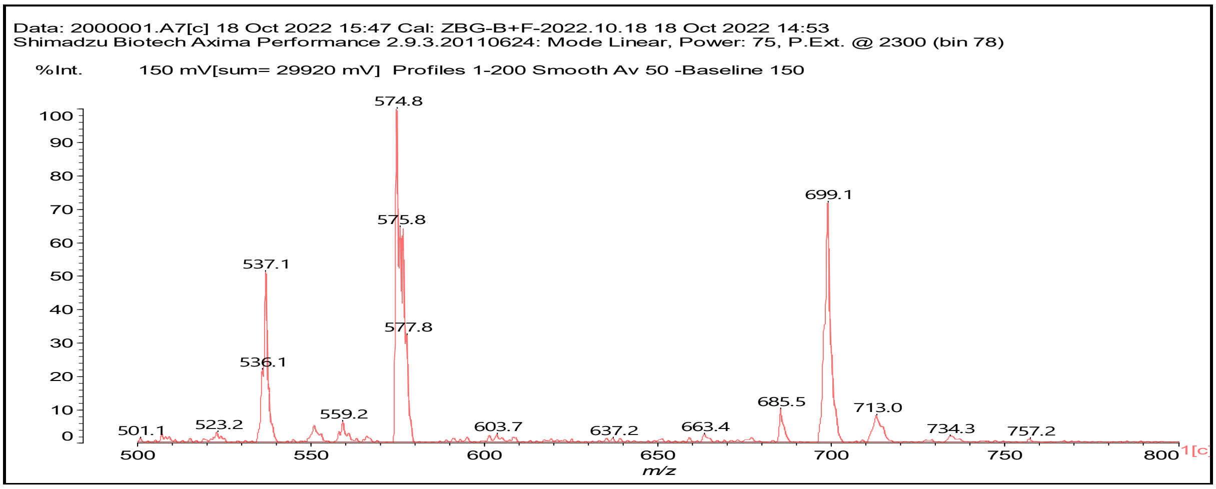

(c)

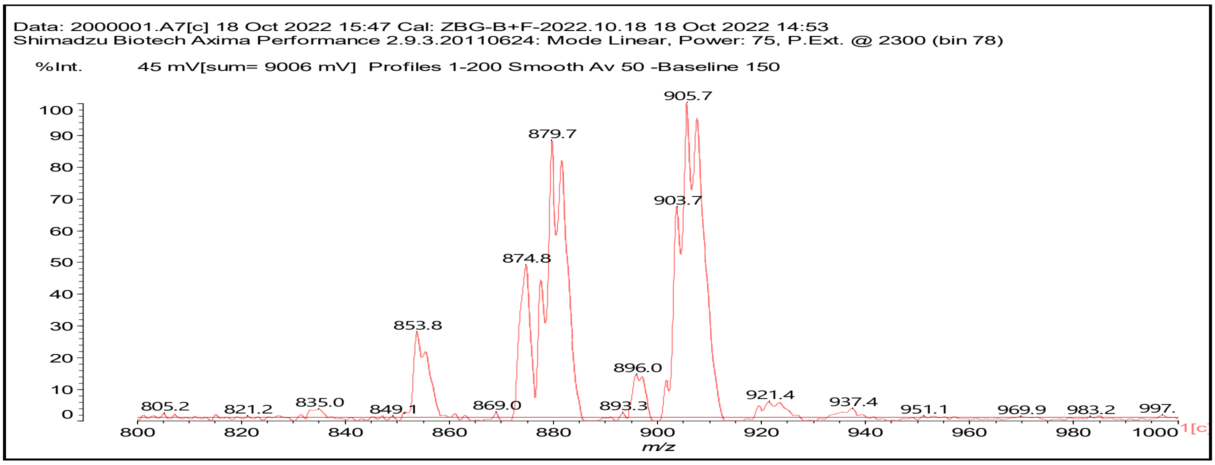

(d)

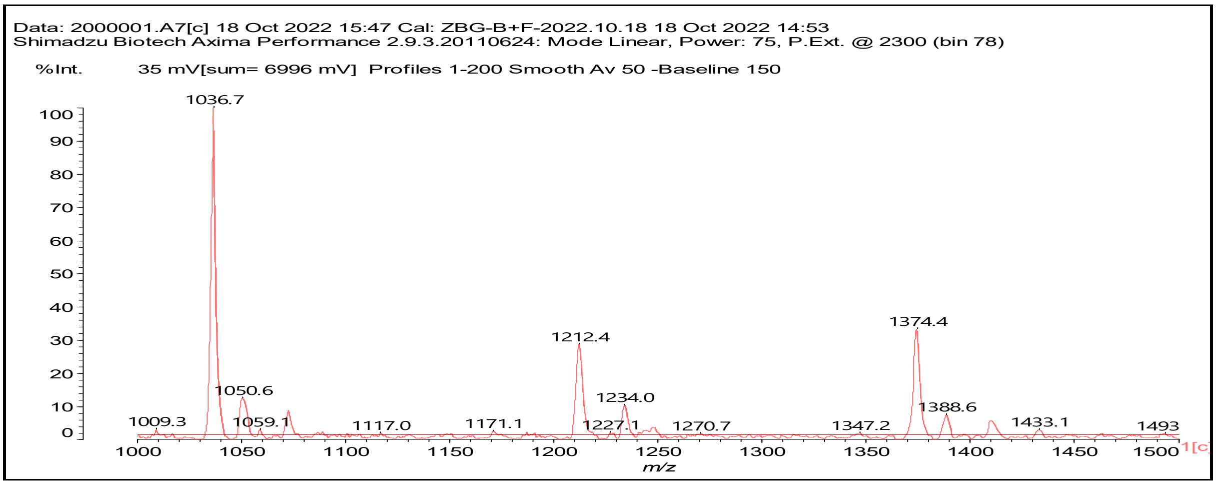

(e)

Figure S3. MALDI ToF spectra of heat-treated beech at 200°C in the (a) 20-1000 Da range and details of the (b) 20-500 Da range, (c) 500-800 Da range, (d) 800 1000 Da range, (e) 1000 -1500 Da range.

Smaller molecular weight oligomers are in greater majority at 200°C than at 300°C, meaning that furanics are already generated, they oligomerize but not yet to very high molecular weight and cross-linking, but are less cross-linked.

Table S1. Assignement of species to the MALDI ToF peaks for beech wood heat treated at 200°C after heat treatment.

---

177 Da = no Na+, with 176 deprotonated, 178 normal (Calc 178 Da

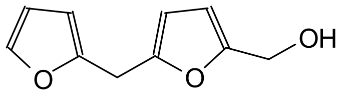

199 Da = with Na+, deprotonated, calculated 199 Da

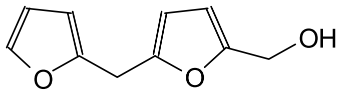

324-326 Da = no Na+, same species as for the 300°C, but bigger peak that at 300°C

361 Da = with Na+, same species as for the 300°C, but bigger peak that at 300°C

537 Da = with Na+, same species as for the 300°C,

574 Da = with Na+, same species as for the 300°C,

853 Da =with Na+, same species as for the 300°C

904-907 Da = no Na+, same species as for the 300°C

1037 Da = with Na+, same species as for the 300°C

1151 Da =with Na+, same species as for the 300°C

MALDI ToF SPECTRA OF BEECH WOOD TREATED AT 103°C

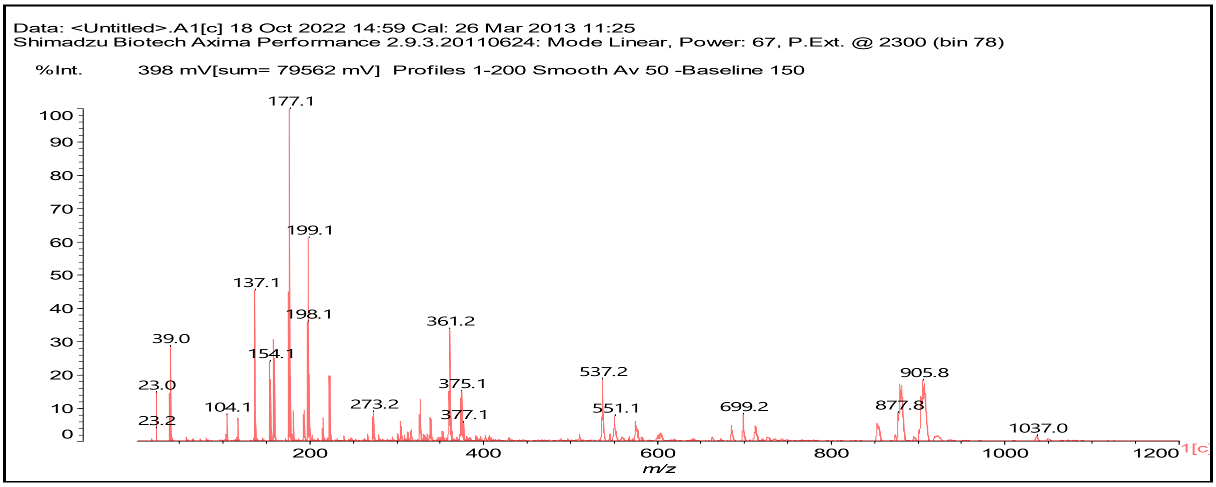

(a)

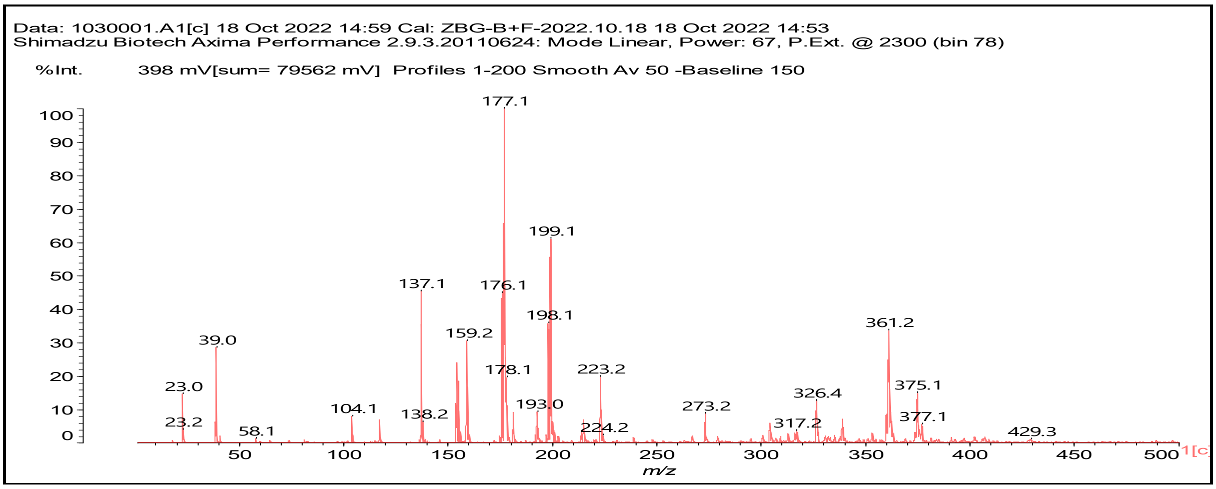

(b)

Figure S4. MALDI ToF spectra of heat-treated beech at 103°C in the (a) 20-1200 Da range and details of the (b) 20-500 Da range.

The low molecular weight 177 Da species (furfural) predominates. The 574 Da species is practically non-existant, and the 537 Da and 551 Da are small, thus very little cross-linked furanics occurs at 103°C, but the species at 907 Da already exists indicating that linear polymerization occurs already, but very little cross-linking. Cross-linking increases progressively from 103°C (very low) to 200° (it starts to be significant but small), to 300°C (considerable cross-linking).

COMPARISON OF MALDI ToF SPECTRA OF FIR WOOD TREATED AT 200°C AND 300°C

FIR WOOD MALDI at 300°C

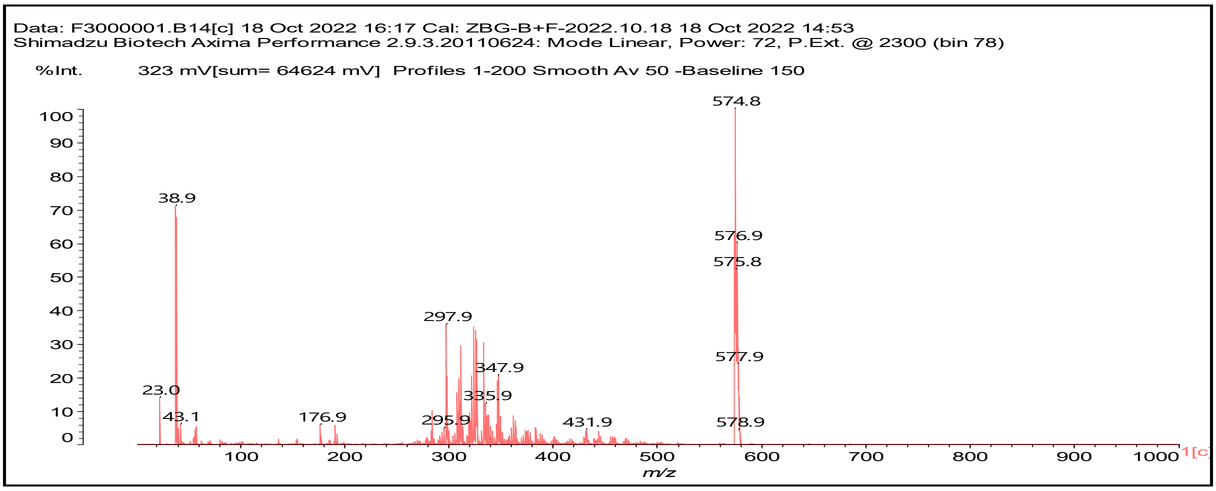

(a)

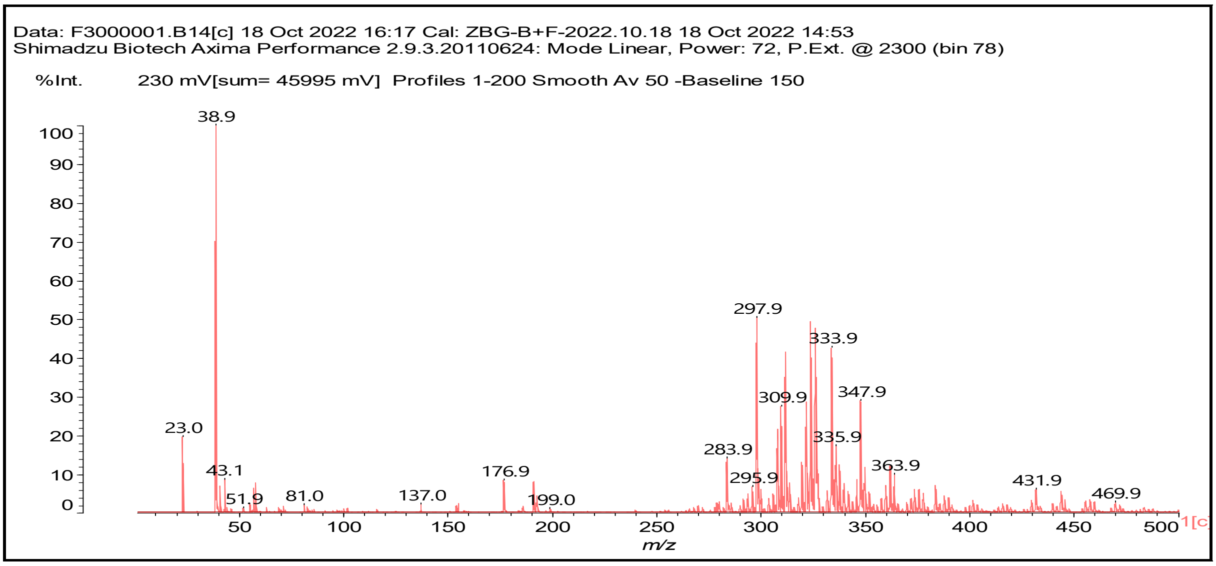

(b)

Figure S5. MALDI ToF spectra of heat-treated fir wood at 300°C in the (a) 20-1000 Da range and in the (b) 20-500 Da range.

At 300°C fir wood is strongly and mostly cross-linked with the cluster at around 574 Da totally predominating. The lower molecular weights and linear oligomers are in great minority (only the 177 Da is present and is very small) and all the others are absent. There are some fragments of linear oligomers in the cluster around 324 Da (one can see the 324 Da no Na+, and the 347 Da with Na+), but that is all. The furanics are more cross-linked than in beech.

FIR WOOD MALDI at 103°C

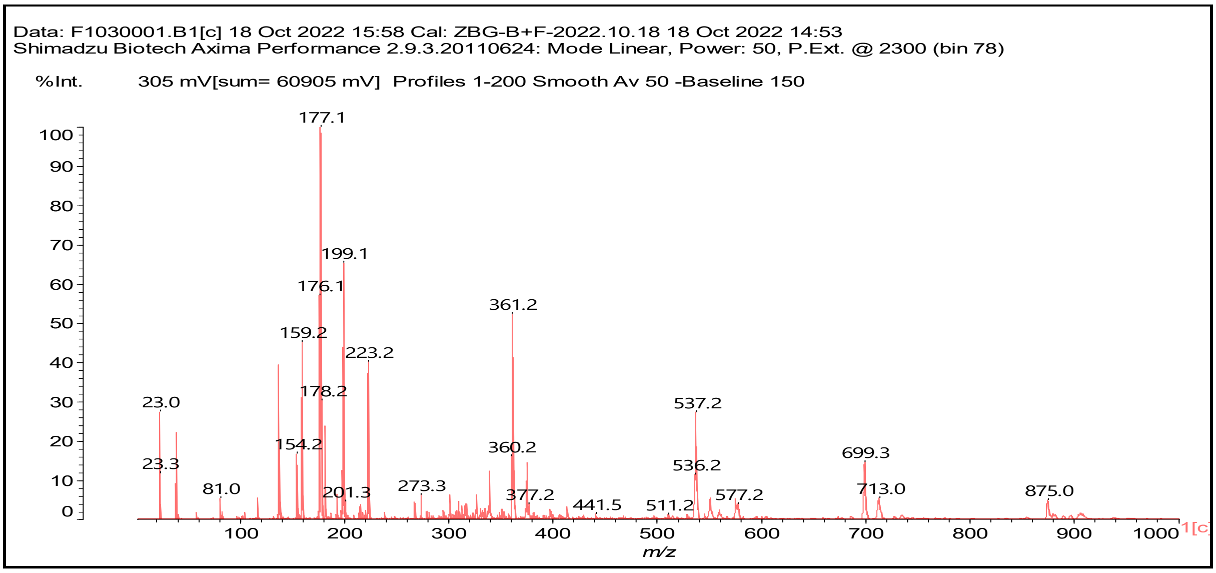

Figure S6. MALDI ToF spectra of heat-treated fir wood at 103°C in the (a) 20-1000 Da range.

At 103°C the small linear species predominate and there is some start of cross-linking (the 537 Da peak) but at the smaller molecular weights. It means that the furanic oligomers have formed but at small molecular weight and predominantly linear.
